# Critical reflections and reflexivity on responding to the needs of LGBTQ+ youth in a global pandemic

**DOI:** 10.1177/1473325020981080

**Published:** 2021-03-04

**Authors:** Gio Iacono, Shelley L Craig, Rachael Pascoe

**Affiliations:** School of Social Work, University of Connecticut, Hartford, USA; Factor-Inwentash Faculty of Social Work, University of Toronto, Toronto, Canada

**Keywords:** LGBTQ+ youth, critical reflexivity, pandemic-related disparities, affirmative practice, cognitive behavioral therapy

## Abstract

The global community has been significantly impacted by the COVID-19 global pandemic. LGBTQ+ (i.e., lesbian, gay, bisexual, transgender, queer, etc.) youth may face increased stressors amidst the pandemic given their significant mental and sexual health disparities, pervasive rejection — including quarantining in homes with heightened risk of abuse and victimization, and a lack of access to essential resources. Responsive supports are needed at this time for vulnerable LGBTQ+ youth, particularly tailored mental health supports. This critical reflexive paper will highlight, as qualitative social work researchers and practitioners, the swift response to the needs of vulnerable LGBTQ+ youth across Canada during this pandemic. We provide a transparent account of how we have utilized critical reflexivity, cultivated through qualitative research, to support LGBTQ+ youth. This article will elucidate the importance of critical reflexivity in effectively transitioning essential offline mental health services for LGBTQ+ youth to a technology-mediated mental health affirmative intervention. The aim of this paper is to provide qualitative researchers and practitioners practical direction through important insights gleaned by supporting marginalized LGBTQ+ youth during particularly trying times such as a global pandemic.

COVID-19 has greatly impacted the global community in unprecedented ways. There have been countless news stories of great panic, calamity, and fallout; as well as courage, hope and inspiration. We have witnessed brave front-line social workers offering critical support and strength to communities, and the wielding of collective action and opportunities to advocate for important social changes (e.g., universal basic income; racial justice efforts) and access to essential resources. LGBTQ+ (i.e., lesbian, gay, bisexual, transgender, queer, etc.) communities across the globe, particularly those most marginalized members, may face unique and disproportionate physiological, psychological, social, and financial challenges amidst this global pandemic (Human Right Campaign Foundation; HRCF, 2020a). For instance, recent research in the US reveals that LGBTQ+ communities are more likely to have experienced a cut in work hours compared to the general population, and are twice as likely to believe their finances will be worse off in one year compared to the general population (HRCF, 2020b).

LGBTQ+ youth may face heightened stress amidst the COVID-19 pandemic given their well-documented mental and sexual health disparities (e.g., depression, suicidality), familial and peer rejection, and a lack of access to resources due to their age and pervasive discrimination (Meyer, 2003; Russell and Fish, 2016; Taylor et al., 2011). For instance, while it is estimated that 7% of youth in the US are LGBTQ+, 40% of homeless youth are LGBTQ+ identified (Fowlkes, 2020; True Colors United, 2020), making it extremely difficult to protect themselves (e.g., via social distancing, personal protective equipment) from COVID-19. Additionally, LGBTQ+ youth may need to quarantine in hostile family situations where they may experience abuse and victimization due to their sexual and gender minority identities (HRCF, 2020a). All of these factors may disproportionately impact the wellbeing and mental health of vulnerable LGBTQ+ youth across the globe.

Proactive responses are required to support LGBTQ+ youth during COVID-19. Specifically, tailored mental health supports may provide a foundation to adequately address the needs of the most vulnerable LGBTQ+ youth during a pandemic. As social work researchers and practitioners, who were already responding to the needs of LGBTQ+ youth offline in the community through the implementation of affirmative cognitive-behavioral group interventions (i.e., AFFIRM) (Craig et al., 2019) , we decided to swiftly respond to the needs of vulnerable youth by offering AFFIRM online to LGBTQ+ youth across Canada.

While this response came with much anxiety, given the difficult reality of the newly announced pandemic, we knew that responding to the needs of vulnerable LGBTQ+ youth would be worth grappling with the uncertainty and extra work that this approach would bring. In doing so, we have been able to mitigate the risks of discontinuing essential mental health services to vulnerable youth who would otherwise potentially find themselves in dire circumstances (e.g., extreme isolation, suicidality). In our team discussions at the beginning of the pandemic, we discussed the risks and benefits of providing a familiar service, with an unfamiliar online platform. We were grappling with many of the same challenges faced by LGBTQ+ youth (e.g., uncertainty about the future, isolation), and it was together that we could discuss those feelings and experience support. It has been profoundly meaningful to support LGBTQ+ youth through a research project evaluating a technology-mediated mental health group intervention, AFFIRM (Craig et al., 2019). This approach has resulted in improved mental health and coping skills, and an increased sense of social support among LGBTQ+ youth (Craig and Austin, 2016). As LGBTQ+ identified social work clinician-scholars, we have utilized reflexivity skills cultivated through qualitative research during this pandemic, as we have been challenged to be reflective in our support of our communities. Ultimately, we felt that the benefits of providing an immediate service greatly outweighed the potential risks of meeting with youth online while they were quarantining. In demonstrating the value of reflexivity in effectively transitioning AFFIRM to an online platform to support LGBTQ+ youth, the following sections aim to provide important insights that we have gleaned from supporting a particularly marginalized population globally, LGBTQ+ youth.

## Working through feelings of helplessness

There have been times during self-isolation where our desires and efforts to help ourselves were thwarted by extreme anxiety; these experiences at times eroded our sense of efficacy in being able to best support LGBTQ+ youth. Our desire to support LGBTQ+ youth was strong, but often imbued with a sense of felt helplessness, given the barriers they faced both during the quarantine and as a marginalized group (e.g., not being out at home, experiencing crises, intrafamilial victimization). We knew through countless prior clinical assessments and research activities that the youth we worked with presented with many psychosocial risks. We felt that we had to process our reflections, which we did in our meetings, in order to funnel our feelings towards meaningful action, and attempt to be helpful and present for the LGBTQ+ youth we served. Although we all felt apprehensive, we were also confident in our abilities, and steadfast in our need to respond.

Skillful reflexivity necessitates practice (Markham, 2017). Thus, critical reflexivity was fostered by carving out a regular time in our day to ask ourselves difficult questions about our work with LGBTQ+ youth during a pandemic. We asked ourselves: How do we stay grounded in this work while supporting youth?; Were we making a difference?; How do we know they are truly supported and safe?; How do we stay motivated when we are dealing with our own stressors and anxiety? Additionally, we carefully considered whether we were going to be able to effectively mitigate the risks of providing mental health services to a youth population with many risk factors (e.g., elevated levels of suicidality), particularly when the literature calls for offline approaches when working with high-risk populations. How will we manage our own human reactions, including stress and fear? What practices did we need to engage in to feel regulated? These, and many more related questions created much stress — and increasing clarity — as we moved through our decision-making process and weighed the benefits and risks.

What we discovered as we established this reflexive stance was that we needed to muster up the courage and strength to respond to the emerging needs of the youth we serve. We needed grit, tenacity, and compassion — for ourselves and for LGBTQ+ youth — to move past fear and apprehension and show up as supportive social work professionals. Ultimately, it took a combination of critical reflection, meditation, clinical consultation and deepened communication to finally tap into an inner source of energy to be present for the LGBTQ+ youth that we wanted to support through this pandemic.

## Positionality and social locations

In reflexively practicing qualitative social work inquiry, it is critical to identify how our frame of reference is situated (i.e., within a particular local, political, and historical context) (Probst and Berenson, 2014). This approach is important in helping us determine how our social positions, identities, places of privilege and disadvantage impact the work we do with LGBTQ+ youth. A critical piece in doing this work, moving all mental health services online to support young LGBTQ+ folks, required revisiting our positionalities within the context of a global pandemic. How am I, coauthor GI, located and positioned within various social, historical, political contexts that inform how I am affected by the pandemic? How do my social locations (GI) as a white, cisgender, male-identified, queer person, that conducts social work research and practice impact my various privileges and buffer against stressors in this situation? How do my cultural experiences being a child of immigrants who fled precarious situations, post-World War II, contribute to my hypervigilance during this time and my ability to be present and support vulnerable LGBTQ+ youth? I continue to grapple with these questions. What has been interesting though, since moving all services virtually, is that deep relationships have formed among LGBTQ+ youth AFFIRM participants, and the feared risks of not being able to effectively support vulnerable youth on a virtual platform, in a pandemic, were unfounded. In fact, I (GI) pleasantly discovered rich and durable displays of youth participants’ resilience in this pandemic, leaving me completely amazed in witnessing the power of the human spirit to overcome adversity.

Coauthor RP, as well, wondered how my privileges and disadvantages played a role in my approach to offering support for LGBTQ+ youth during the COVID-19 pandemic. As a white, queer, ciswoman with straight privilege, in academic research and social work clinical practice positions, I had to grapple with what support I could offer vulnerable youth during this time. I (RP) have also recognized that my own anxieties and my family’s cultural history of intergenerational trauma, which included facing antisemitism and searching for safety after World War II in Canada, have affected me during this pandemic. At times, my hypervigilance and awareness of danger were not wholly attributed to the present situation, but rather the real dangers that my family experienced and the fear that was passed on to me. As the pandemic continued and I continued to facilitate groups, I noticed strengths and tensions arise in the context of online groups. As a social worker, I felt it was important to help all group members, but soon learned that the online modality was not suitable to everyone who expressed interest. This allowed me to practice letting go, and come to terms with the fact that I cannot help everyone expressing interest in AFFIRM at this time. Like my cofacilitator (GI), I was also pleasantly surprised that online therapy reportedly was effective, and the same experiences of connection, community, and support experienced when groups are offered in person were still shared by the members of the groups during their final sessions. As well, I was pleasantly surprised by the glimpses into the group members’ homes and lives. What I would previously consider disclosures of my life, such as my cat walking across the screen, became moments of shared humor and deeper connection, eliciting sharing of pets, panel shots of group members’ bedrooms, and demonstrations of meaningful trinkets.

As a white, cisgender lesbian academic with decades of experience working with LGBTQ+ youth, that is also the PI on the AFFIRM study, this coauthor (SLC) balanced anxiety with pragmatism. She was fearful of the impact that COVID-19 was having on our LGBTQ+ participants, yet recognized that we had to be nimble and immediately respond to provide support. The concerns were exacerbated when she recognized that utilizing a new technology to engage youth in crises would contribute to additional stress on the AFFIRM facilitators, and she struggled with whether she was providing enough support to facilitators. She was also worried that her grasp of the technology would hamper her support of the facilitators. In addition, she had to consider ways to integrate the new reality of the pandemic into the research, while maintaining the study quality and minimizing participant stress. These critical reflections/questions generate an awareness that can inform the way we work with youth and allows for a critical analysis in our practice that contributes to our growth as researchers.

As social work professionals, being ‘reflexive’ is not necessarily about specific activities but rather an attitude and openness to maintain “self-awareness and agency within that self-awareness… to think about our thinking and our feeling, to have a feeling about a feeling, to have a desire about a desire, and that this self-awareness flows into action” (Rennie, 2004: 183). As illustrated below, we have integrated the knowledge gained through our reflexivity into actions to better support LGBTQ+ youth in a pandemic.

## Showing up and being effective with LGBTQ+ youth during a pandemic

### Meeting where they are at

We have found that in order to delve deeper into any challenge youth were experiencing (e.g., unsafe living conditions, suicidality) we needed to support them to practice self-reflection before problem solving and taking action steps. An important element of this work with LGBTQ+ youth first involved supporting them *where they are at* in terms of any immediate stress and anxiety they were experiencing. For instance, while AFFIRM consists of planned check-in questions/activities, we needed to adapt our check-ins and take more time to process their pandemic-related stressors. Additionally, we needed to utilize increased grounding approaches (e.g., deep breathing, mindfulness) to support meeting the goals for the session. Once we have engaged youth in grounding and processing, we move to remind them of previous session content and skills, current session materials, and begin to adequately respond to their overall needs.

### Transparency and vulnerability

An interesting element of this work involves the explicit and increased use of self and vulnerability. As social work professionals, we are not immune to pandemic-related stressors. We have found it helpful to increase personal transparency to normalize and validate the youth experience. For example, in response to youth’s struggles with regulating their sleep schedules, finding the motivation to do schoolwork, and losing touch with once close friends, we responded with empathy and useful therapeutic self-disclosure around our own struggles with the same issues. We have seen the value of vulnerability, as queer people ourselves, in sharing with younger people in our community the sense of shared humanity. They may see that, as queer people, we are struggling with stress and also finding ways to cope and be resilient in the face of uncertainty and disruption. We believe the extra vulnerability also contributes to the lessening of power differentials (e.g., inherent researcher-participant and client-therapist power imbalances), which has been very well received among youth whose marginalized identities renders them with less power in many social contexts.

### Going the extra mile

We have also had to offer wraparound support to LGBTQ+ youth while they participate in AFFIRM online. For instance, we have extended our scope of support to offer case management, referrals (housing, food banks, legal), as well as additional text/phone crisis support. Typically, these extra supports would be provided by the collaborating agency in which AFFIRM would be held. As a geographically-diverse research project, we have partnered with various agencies to provide LGBTQ+ youth local resources in addition to effective mental health treatment offered through AFFIRM. Additionally, we have aimed to support one another as LGBTQ+ social work researchers and clinicians. For instance, our communication and consultation with one another has significantly increased (e.g., increased processing and debriefs post-session; greater online social time and informal text-messaging) to ensure that we remain reflexive in this important work, and are providing the best possible service to LGBTQ+ youth.

## Conclusion

As LGBTQ+ researchers and clinicians, we have been profoundly moved by the need among vulnerable LGBTQ+ youth during the pandemic We have responded by offering support during a difficult, uncertain and unprecedented time. While we may regard this period as a global *health* crisis, for marginalized communities such as LGBTQ+ youth, the crisis is significantly more extreme given the real intersectional mental health, social, cultural, and institutional barriers they regularly face in society. LGBTQ+ youth have reported to value our vulnerable support. By responding to LGBTQ+ youth’s desire for immediate and comprehensive support during our research projects, and offering them the space to process difficult emotions, we have cultivated our own reflexivity and contributed to our own wellbeing as well as that of our LGBTQ+ youth participants.
